# E-liquid flavor alters nicotine exposure, puff topography, and subjective effects under *ad libitum* use conditions

**DOI:** 10.1016/j.abrep.2026.100665

**Published:** 2026-01-06

**Authors:** Arit M. Harvanko, Conor A. Ruzycki, Jacob McDonald, Eric D. Claus, Megan Schroeder, Carolina Ramôa

**Affiliations:** a Food and Drug Administration, Center for Tobacco Products, Office of Science, 10903 New Hampshire Avenue, Building 71, Room G335, Silver Spring, MD 20993-0002, United States; b Lovelace Biomedical Research Institute, 2425 Ridgecrest Dr. SE, Albuquerque, NM 87108-5127, United States; c The Pennsylvania State University, 201 Old Main, University Park, PA 16802, United States

**Keywords:** Behavioral pharmacology, Electronic cigarette, ENDS, Clinical pharmacology, Flavors

## Abstract

**Introduction:**

Electronic nicotine delivery systems (ENDS) are most often used with e-liquids having fruit, and menthol or mint flavor, with tobacco flavor being least prevalent. This study evaluated the effect of a tobacco, fruit, and menthol e-liquid flavor on ENDS abuse liability.

**Methods:**

Fifty-two regular ENDS users participated in a double-blinded, randomized, crossover-design study. Study conditions were ENDS (KangerTech CUPTI) with tobacco, menthol, or fruit flavor e-liquids with 1.2 % (all flavors) or 0 % (fruit only) freebase nicotine concentration. On separate days participants used the study products for 10 prescribed puffs and 1 h of *ad libitum* use. Plasma nicotine concentration, puff topography, and subjective effects were measured.

**Results:**

Following *ad libitum* use, tobacco flavor ENDS were associated with significantly lower plasma nicotine levels than other nicotine-containing ENDS. Significantly fewer puffs were taken from the tobacco flavor ENDS than other products, and the 0% nicotine fruit flavor ENDS was associated with greater puff duration and puff volumes than other products. Tobacco flavor was rated significantly lower than other flavors on product liking (e.g., “tastes good” or “pleasant”), while only the 0% nicotine fruit flavor ENDS was associated with significantly greater withdrawal symptoms (e.g., ENDS craving, or urge to use an ENDS) compared to the other flavors.

**Conclusions:**

Tobacco flavor ENDS were rated lower on liking, and associated with less nicotine exposure and fewer puffs taken, but comparably attenuated withdrawal symptoms to other flavor ENDS, while the non-nicotine fruit flavor did not affect withdrawal symptoms.

## Highlights

• Tobacco flavor had lower liking and nicotine exposure than other e-liquids.
• Tobacco flavor eased withdrawal less than menthol or fruit flavor e-liquids.
• A zero-nicotine fruit e-liquid did not ease withdrawal symptoms.
• Tobacco flavor e-liquids may have lower abuse liability than non-tobacco flavors.
• E-liquids with lower liking and nicotine exposure may substitute for cigarettes.

## 1 Introduction

Electronic nicotine delivery systems (ENDS) are typically used with fruit, menthol, or mint flavored e-liquids, with tobacco flavor being least common (Bansal-Travers et al., 2024). Widespread availability and popularity of non-tobacco ENDS flavors raise questions about their impact on abuse liability and potential for switching from or reducing combusted cigarette (CC) use. ENDS flavor could alter user behavior (i.e., puffing topography), also affecting nicotine exposure and abuse liability. and alter subjective effects like satisfaction and craving relief, which could affect the likelihood of switching from CCs to ENDS and of ENDS addiction.

Previous research on the abuse liability of ENDS flavors has yielded mixed findings and some methodological limitations. While some studies have demonstrated that sweet or fruit flavors can increase nicotine exposure compared to tobacco flavors (St Helen, Dempsey, Havel, Jacob, & Benowitz, 2017) and enhance subjective appeal and reinforcing value (Audrain-McGovern et al., 2016, Goldenson et al., 2016), these studies have been limited by small sample sizes (n = 14–32), and lack of nicotine-free control conditions. Additionally, results have been inconsistent regarding subjective effects, with some studies finding no significant differences in satisfaction between flavors despite differences in nicotine exposure (St Helen et al., 2017). These methodological limitations and mixed findings highlight the need for more rigorous experimental designs to understand how flavors influence ENDS use patterns and abuse liability.

The current study addresses these gaps by incorporating a larger sample size, a nicotine-free control condition, and a randomized, double-blinded, crossover design to assess effects of ENDS flavor on nicotine exposure, puffing behavior, and subjective liking and withdrawal symptoms following *ad libitum* use. Study findings enhance understanding of how ENDS flavors affect abuse liability under controlled conditions within a clinical setting, providing important evidence for the potential role of ENDS in switching from CCs given the substantial public health implications with current U.S. adult CC and ENDS use rates of approximately 8 % and 4.1 %, respectively (Arrazola, Husten, Cornelius, & Armour, 2025).

## 2 Methods

### 2.1 Participants

Recruitment criteria included: age 18–55, regular nicotine-containing ENDS use (≥ 25 days in the past month), CC use ≤ 1 day/week in the past 30 days, no current smoking cessation treatment, and exhaled carbon monoxide < 6 ppm. Participants were excluded if they reported illicit drug use in the past 30 days but could report currently using other tobacco products. During 2018–2020, 307 participants were screened by telephone, 89 completed an in-person screening visit and 61, 52, 49, and 46 completed one, two, three, and all four study days, respectively. Participants were compensated up to $875 ($75 for screening and $200 per exposure visit). All participants provided informed consent. Only those who completed at least two study visits were included in the final dataset. This study was approved by an external institutional review board (Advarra: #Pro00036406).

### 2.2 Study products

Participants used a KangerTech CUPTI™ ENDS at 35 W, with a 5 mL tank and 0.5 O 316 L coil. This ENDS type was popular when this study began (2018).

E-liquids were selected based on manufacturer-reported popularity in tobacco, menthol, and fruit flavor categories and purchased from Avail Vapor, LLC. Five e-liquids were used: one for the initial screening/practice day (Tobacco Row [tobacco flavor] with 1.2 % nicotine concentration), and four for exposure days (Jamestown Tobacco [tobacco flavor], Arctic Blast [menthol flavor], Mardi Gras [fruit flavor] with 1.2 % nicotine concentration, and Mardi Gras with 0.0 % nicotine concentration). All e-liquids contained 70:30 propylene glycol:vegetable glycerin.

### 2.3 General procedures

The study included an initial screening/practice day and four exposure days separated by 48 h to 10 business days. At the screening/practice day, participants provided informed consent and completed a demographics and tobacco use history questionnaire and measures of nicotine dependence, including modified versions of the Fagerström Test for Nicotine Dependence (Heatherton, Kozlowski, Frecker, & Fagerstrom, 1991) and Penn State Electronic Cigarette Dependence Index (Foulds et al., 2015). Past 30-day tobacco use was assessed using the Timeline Follow-back method (Sobell & Sobell, 1992) and confirmed via urinary cotinine of ≥ 100 ng/mL. At the screening/practice day, participants completed a prescribed puffing session with Tobacco Row 1.2 % e-liquid to familiarize themselves with the study ENDS and procedures.

Exposure days were randomized according to a Williams Design generated with SAS v. 9.4 (SAS Institute Inc., Cary, NC). Participants were asked to abstain from ENDS for 12 h and all other tobacco products for 72 h, prior to each exposure day (verified post-hoc using plasma nicotine levels). Each exposure day included a prescribed and *ad libitum* use session. During the prescribed use session participants took 10 directed puffs with 30 s inter-puff intervals, followed by a 115-minute observation period. Only *ad libitum* use is included in this report.

During the *ad libitum* session (121 min following initiation of prescribed use), participants used the study ENDS for 1 h. One participant stopped using the study ENDS before the *ad libitum* session completed; they remained in the facility and remaining measures were taken and blood samples collected. Blood samples were collected, and heart rate and blood pressure were measured at the beginning, end, and 1 h following the *ad libitum* session. Within 5 min following the end of the *ad libitum* session, blood pressure and heart rate were measured and participants completed the Direct Effect of Nicotine Scale, Direct Effects of E-cigarettes Scale, Drug Effect Scale, and Abstinence Symptom Suppression Scale. Within 30 min following the *ad libitum* session, participants completed the Minnesota Nicotine Withdrawal Scale (MNWS) and Questionnaire of Smoking Urges – Brief (QSU-B). Where appropriate, questionnaires were adapted for ENDS by replacing the word “cigarette(s)” with “e-cigarette(s).”.

### 2.4 Study measures

#### 2.4.1 Plasma nicotine

Plasma nicotine concentrations were measured with gas chromatography–tandem mass spectrometry/mass spectrometry. The limit of quantitation for plasma nicotine concentration was 2.0 ng/mL.

#### 2.4.2 Puff topography

The Clinical Research Support System (CReSS Pocket, Version 3, Borgwaldt, KC) measured puff topography (i.e., puff number, duration, and volume, average flow rate, and inter-puff interval).

#### 2.4.3 Subjective effects

Six subjective effects measures were administered. The following were 100-point visual analog scales (0=“not at all”, 100=“extremely”): Direct Effect of Nicotine Scale (10-item scale that measures nicotine effects), Direct Effects of E-cigarettes Scale (10-item scale that measures ENDS effects), and Abstinence Symptom Suppression Scale (11-item scale that measures nicotine or ENDS abstinence symptoms). The Drug Effect Scale includes five-items related to product liking rated on a 5-point Likert scale ranging from “not at all” to “extremely.” Differences in MNWS (Hughes & Hatsukami, 1986) and QSU-B (Tiffany & Drobes, 1991) total scores were examined from preceding to following the *ad libitum* session.

### 2.5 Statistical analysis

Plasma nicotine concentration values were baseline corrected using the elimination rate constant (Kel) calculated from the participant’s own data: C(t)adjusted=C(t)observed-C(0)e-Kel(t), where t is the time point, 0 is the baseline time point, C(t)adjusted is the adjusted concentration at a time point, and C(t)observed is the observed concentration at a time point. Two participants had quantifiable plasma nicotine concentrations at Mardi Gras 0.0 % ENDS sessions and were removed from analyses for that session.

Statistical comparisons of plasma nicotine parameters included area under the plasma nicotine concentration–time curve (AUC_0-60_) and maximum plasma nicotine concentration (C_max_). Comparisons were conducted with a linear mixed model to include data from participants with fewer than four exposure visits. Sequence, visit number, and product were fixed effects, and participants were a random effect. Nicotine exposure parameters were log-transformed for analysis. Analyses were performed using SAS v. 9.4 and α = 0.05.

## 3 Results

Participant (*n* = 52) demographics and tobacco use are reported in Supplementary Table 1. Participants reported using ENDS an average of 3.1 times per day (SD = 1.6) for an average of 3.7 years (SD = 2.4), typically using e-liquids with a nicotine concentration of 14.1 mg/mL (SD = 28.6) and nearly all participants (98 %) used rechargeable ENDS. Other tobacco product use was minimal; two reported current cigar use (< 2x/month) and three reported hookah use (≤ 4x/month).

### 3.1 Nicotine exposure

Table 1 presents nicotine exposure parameters. Jamestown Tobacco 1.2 % was associated with significantly lower plasma nicotine C_max_ and AUC_(0-60)_ relative to other nicotine-containing ENDS (*p*’s < 0.001).

**Table 1:** Plasma nicotine exposure parameters.

	Mardi gras (0 %)	Arctic blast (1.2 %)	Jamestown (1.2 %)	Mardi gras (1.2 %)	Main effect	Post-hoc comparisons
**C_max_,** ng/mL	BLQ	4.58 (2.83)	2.65 (2.99)	5.09 (2.84)	F = 54.34^***^	Arctic Blast & Mardi Gras 1.2 % > Jamestown^***^ All 1.2 % > Mardi Gras 0 %^***^
**AUC,** ng/mL min	BLQ	494	343	509	F = 40.49^***^	Arctic Blast & Mardi Gras 1.2 % > Jamestown^***^ All 1.2 % > Mardi Gras 0 %^***^

Values are geometric mean (SD of geometric mean) of baseline adjusted plasma nicotine exposure parameters and time to max concentration. BLQ = below limit of quantitation. AUC = area under the plasma nicotine concentration–time curve. ***=*p* < 0.001, **=*p* < 0.01, *=*p* < 0.05.

### 3.2 Puff topography

Supplementary Table 2 lists puff topography variables. Significantly fewer puffs were taken from Jamestown Tobacco 1.2 % relative to all other e-liquids (*p*’s < 0.05). Mardi Gras 0 % was associated with greater puff duration and puff volumes compared to all other ENDS (*p*'s < 0.001).

### 3.3 Subjective effects

**Fig. 1:**
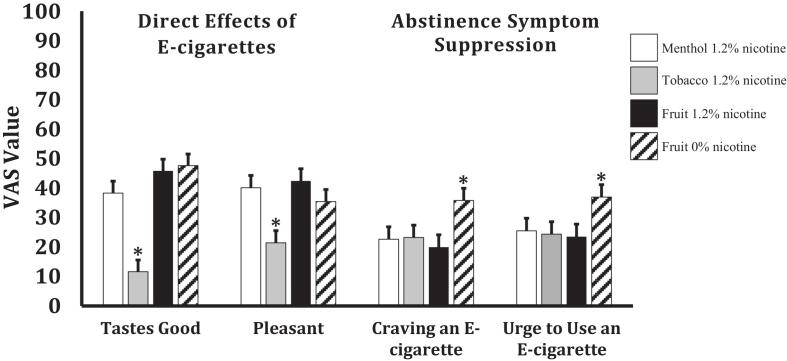
Menthol = Arctic Blast e-liquid, Tobacco = Jamestown Tobacco e-liquid, Fruit = Mardi Gras e-liquid. Visual Analog Scale (VAS) Values are 0–100 from a scale anchored with “Not at all” at 0 and “Extremely” at 100. All values are means with standard errors derived from the linear mixed models. * = Significantly different than all other conditions with *p*’s < 0.05.

Subjective effects results are listed in Supplementary Table 3. Notably, Jamestown Tobacco 1.2 % was rated lower than all other ENDS on several desirable subjective effects, including Pleasant and Tastes Good on the Direct Effects of E-cigarettes Scale (*p*'s < 0.02; Fig. 1) and Enjoy, Taste, Like, and Pleasure on the Drug Effects Scale (*p*’s < 0.001). Mardi Gras 0 %, however, demonstrated less withdrawal symptom alleviation compared to all other ENDS with higher ratings on Use an E-cigarette Right Now on Direct Effects of E-cigarettes (*p*'s < 0.001), lower ratings on Craving an E-cigarette (Fig. 1), Impatient, and Urge to Use an E-cigarette on Abstinence Symptom Suppression Rating (*p*’s < 0.05), and less reduction in urges compared to all other ENDS on the QSU-B (*p*’s < 0.02).

## 4 Discussion

Results showed that e-liquid flavor affected nicotine exposure, puffing topography, and ratings of desirable subjective effects (e.g., product liking and satisfaction), but not reports of withdrawal symptom alleviation. The tobacco flavor was associated with fewer puffs and subsequent lower nicotine exposure, perhaps because of lower desirable subjective effects. This suggests flavor-related subjective effects can influence behavioral patterns and resulting pharmacological outcomes. Despite reduced nicotine exposure from the tobacco flavor, and regardless of flavor, the presence of nicotine was the primary driver of withdrawal symptom relief. This suggests that, while flavor affects appeal and use patterns, nicotine content remains the key factor for addressing tobacco dependence symptoms.

These results are consistent with previous research demonstrating that a fruit flavor e-liquid was associated with greater nicotine exposure than a tobacco flavor e-liquid, while ratings of tobacco withdrawal symptoms were not significantly different (St Helen et al., 2017). Although participants in that study (St Helen et al., 2017) did not rate a tobacco flavor significantly lower than a fruit flavor (i.e., strawberry) on measures of subjective liking, participants in the current study rated the tobacco flavor lower on measures of taste and liking; nonetheless, ratings of withdrawal symptoms were not significantly different. Our results are partially consistent with another study that found a tobacco flavor e-liquid, relative to a preferred non-tobacco flavor e-liquid, was rated lower on subjective liking, though it did not significantly differ on measures of nicotine exposure (Vargas-Rivera et al., 2020). The current study demonstrates that, even if nicotine exposure and subjective ratings of desirable effects (e.g., liking) are lower for a tobacco flavored e-liquid, management of tobacco withdrawal symptoms appears driven by presence of nicotine in the e-liquid, and not associated with subjective liking. These results suggest tobacco flavored e-liquids may be useful for switching from CCs because they address withdrawal symptoms; however, lower desirable subjective effects from tobacco flavors could limit complete switching as CC users may choose fruit or menthol flavored ENDS for greater desirable subjective effects. Therefore, non-tobacco flavors could facilitate greater switching but potentially increase ENDS dependence risk from greater use.

Limitations of this study include: 1) this study only tested popular examples of flavors from three categories (i.e., tobacco, menthol, fruit) and although data on participants’ preferred e-liquid was captured, many e-liquids could not be grouped into these categories given labelling and naming that did not provide clear categorization; as a result personal preference for certain types of flavors was not accounted for in this study, 2) although the study ENDS had a nicotine concentration consistent with participants’ usual e-liquids (i.e., 14.1 mg/mL) the study ENDS with 1.2 % nicotine concentration may not generalize to higher nicotine concentrations and other types, 3) the predominantly white male sample and adult age range (ages 18–55) may limit generalizability to people of other races, female users, and adolescents (who are more likely to use flavored ENDS products), and 4) use in a controlled laboratory environment offers good experimental control but may not reflect real-world use.

## Author statement

All authors have seen and approved the final version of the manuscript being submitted. This article is the authors' original work, hasn't received prior publication, and isn't under consideration for publication elsewhere.

## Data availability

Data from this study are available at: https://doi.org/10.6084/m9.figshare.30757928.

## CRediT authorship contribution statement

**Arit M. Harvanko:** Writing – review & editing, Writing – original draft. **Conor A. Ruzycki:** Writing – review & editing, Methodology, Investigation. **Jacob McDonald:** Writing – review & editing, Supervision, Project administration, Conceptualization. **Eric D. Claus:** Writing – review & editing, Methodology, Formal analysis. **Megan Schroeder:** Writing – review & editing, Writing – original draft, Supervision, Conceptualization. **Carolina Ramôa:** Writing – review & editing, Writing – original draft, Supervision, Conceptualization.

## Funding

This study was funded by the United States Food and Drug Administration, Center for Tobacco Products (HHSF223201310033I).

## Declaration of competing interest

The authors declare that they have no known competing financial interests or personal relationships that could have appeared to influence the work reported in this paper.

## Data Availability

Data will be uploaded following publication to a link included in the manuscript.
