# Supplementary material for: E-liquid flavor alters nicotine exposure, puff topography, and subjective effects under ad libitum use conditions
Source: Addict Behav Rep. 2026 Jan 6;23:100665. doi: 10.1016/j.abrep.2026.100665 (PMC12819110; doi:10.1016/j.abrep.2026.100665)
Supplement: Supplementary Data 1 [file mmc1.docx]

**Supplementary Table 1. Demographic and Tobacco Use Variables**

| **Demographics**  Years of age, *mean (SD)*  Female, *N (%)*  Hispanic, *N (%)*  Race, *N (%)*  American Indian or Alaska Native  Asian  White  Did not specify | 29.6 (10.2)  15 (28.8)  32 (61.5)  5 (9.6)  2 (3.8)  42 (80.8)  3 (5.8) |
| --- | --- |
| **Combusted Cigarettes**  Current combusted cigarette use, *N (%)*  Past combusted cigarette use, *N (%)*  **Dependence** | 2 (3.8)  33 (63.5) |
| Fagerström Test for Nicotine Dependence, *mean (SD)* | 6.2 (2.8) |
| Penn State E-cigarette Dependence Index, *mean (SD)* | 10.6 (4.4) |

**Supplementary Table 2. Puff Topography**

|  | Mardi Gras  (0%) | Arctic Blast (1.2%) | Jamestown (1.2%) | Mardi Gras (1.2%) | Main Effect | Post-hoc Comparisons |
| --- | --- | --- | --- | --- | --- | --- |
| **Puff Duration** (sec) | 3.8 (0.2) | 2.9 (0.2) | 2.8 (0.2) | 3.0 (0.2) | ***F* = 18.96***** | Mardi Gras 0% > All*** |
| **Puff Volume** (ml) | 136.8 (7.7) | 102.8 (7.8) | 96.8 (7.7) | 102.2 (7.7) | ***F* = 14.48***** | Mardi Gras 0% > All*** |
| **Puff Number** | 30.6 (4.1) | 26.7 (4.2) | 17.7 (4.1) | 30.8 (4.1) | ***F* = 4.37**** | All > Jamestown* |
| **Inter-puff Interval** (sec) | 31.8 (10.1) | 46.4 (10.2) | 63.0 (10.3) | 54.8 (10.1) | *F* = 1.91 | - |

All values are mean (standard error). * *p*<.05, ***p*<.01, ****p*<.001. “All” refers to all other conditions.

**Supplementary Table 3. Subjective Effects**

|  | Arctic Blast (0%) | Jamestown (1.2%) | Mardi Gras (1.2%) | Mardi Gras (0%) | Main Effect | Post-hoc Comparisons |
| --- | --- | --- | --- | --- | --- | --- |
| **Direct Effects of Nicotine** |  |  |  |  |  |  |
| Confused | 4.27 (1.33) | 4.22 (1.32) | 5.10 (1.32) | 4.14 (1.31) | *F* = 0.34 |  |
| Dizzy | 10.08 (1.90) | 7.30 (1.86) | 11.20 (1.87) | 5.34 (1.85) | ***F* = 3.37*** | Arctic Blast* & Mardi Gras 1.2%** > Mardi Gras 0% |
| Headache | 6.21 (2.11) | 8.61 (2.06) | 10.06 (2.09) | 7.21 (2.06) | *F* = 0.92 |  |
| Heart Pounding | 5.02 (1.45) | 6.18 (1.42) | 5.67 (1.43) | 4.27 (1.42) | *F* = 0.50 |  |
| Lightheaded | 13.56 (2.52) | 11.40 (2.46) | 18.47 (2.49) | 7.81 (2.46) | ***F* = 4.09**** | Jamestown > Mardi Gras 0%*, Mardi Gras 1.2% > Mardi Gras 0%*** |
| Nauseous | 5.96 (1.68) | 6.73 (1.64) | 6.55 (1.65) | 4.98 (1.63) | *F* = 0.30 |  |
| Nervous | 4.32 (1.41) | 4.05 (1.38) | 6.03 (1.39) | 4.14 (1.37) | *F* = 0.78 |  |
| Excessive Salivation | 5.87 (1.62) | 6.79 (1.59) | 7.01 (1.60) | 6.19 (1.58) | *F* = 0.15 |  |
| Sweaty | 2.52 (1.05) | 4.51 (1.03) | 4.60 (1.04) | 2.91 (1.03) | *F* = 1.82 |  |
| Weakness | 6.27 (1.62) | 5.33 (1.59) | 7.29 (1.60) | 4.22 (1.59) | *F* = 1.15 |  |
| **Direct Effects of E-cigarettes** |  |  |  |  |  |  |
| Satisfied | 43.03 (4.40) | 23.70 (4.33) | 43.21 (4.34) | 32.16 (4.28) | ***F* = 5.47***** | Arctic Blast*** & Mardi Gras 1.2%*** > Jamestown, Mardi Gras 1.2% > Mardi Gras 0%* |
| Pleasant | 40.08 (4.18) | 21.43 (4.11) | 42.39 (4.13) | 35.42 (4.07) | ***F* = 5.67***** | All > Jamestown* |
| Tastes Good | 38.29 (4.03) | 11.60 (3.97) | 45.79 (3.98) | 47.60 (3.92) | ***F* = 20.06***** | All > Jamestown*** |
| Dizzy | 9.93 (1.97) | 6.75 (1.94) | 13.45 (1.95) | 6.29 (1.93) | ***F* = 4.32**** | Mardi Gras 1.2% > Mardi Gras 0%** & Jamestown*** |
| Calm | 55.84 (5.09) | 45.30 (5.02) | 51.08 (5.04) | 48.73 (4.99) | *F* = 1.37 |  |
| Concentrate | 48.77 (4.83) | 41.50 (4.76) | 47.50 (4.78) | 41.33 (4.73) | *F* = 1.33 |  |
| Awake | 52.60 (4.68) | 47.93 (4.62) | 52.30 (4.63) | 45.23 (4.59) | *F* = 1.08 |  |
| Reduce Hunger | 16.31 (3.07) | 17.54 (3.02) | 18.86 (3.03) | 14.89 (3.00) | *F* = 0.55 |  |
| Sick | 8.81 (1.97) | 7.54 (1.92) | 6.93 (1.94) | 3.87 (1.92) | *F* = 1.82 |  |
| Use E-cigarette Right Now | 21.61 (4.16) | 20.22 (4.09) | 19.75 (4.12) | 35.41 (4.09) | ***F* = 7.43***** | Mardi Gras 0% > All*** |
| **Abstinence Symptom Suppression Rating** |  |  |  |  |  |  |
| Anxious | 9.67 (2.04) | 5.39 (2.00) | 8.69 (2.01) | 8.73 (1.99) | *F* = 1.37 |  |
| Craving an E-cigarette | 22.62 (4.23) | 23.24 (4.18) | 19.93 (4.19) | 35.78 (4.15) | ***F* = 5.85**** | Mardi Gras 0 % > All*** |
| Depression/Feeling Blue | 4.52 (1.29) | 4.91 (1.28) | 4.42 (1.28) | 4.29 (1.27) | *F* = 0.08 |  |
| Difficulty Concentrating | 9.23 (2.37) | 6.60 (2.32) | 11.58 (2.34) | 11.38 (2.32) | *F* = 1.68 |  |
| Drowsiness | 16.75 (3.15) | 14.65 (3.10) | 17.02 (3.12) | 16.27 (3.09) | *F* = 0.28 |  |
| Hunger | 21.88 (3.97) | 19.73 (3.91) | 21.11 (3.93) | 23.36 (3.90) | *F* = 0.38 |  |
| Impatient | 11.05 (2.89) | 9.13 (2.83) | 10.81 (2.85) | 18.09 (2.82) | ***F* = 3.47*** | Mardi Gras 0 % > All* |
| Irritability/Frustration/Anger | 7.46 (2.42) | 8.24 (2.37) | 8.58 (2.39) | 13.11 (2.36) | *F* = 1.94 |  |
| Restlessness | 10.24 (2.06) | 8.86 (2.02) | 10.42 (2.04) | 10.52 (2.02) | *F* = 0.27 |  |
| Desire for Sweets | 17.98 (3.58) | 14.28 (3.53) | 15.50 (3.56) | 16.25 (3.53) | *F* = 0.50 |  |
| Urge to Use E-cigarette | 25.48 (4.28) | 24.36 (4.21) | 23.49 (4.24) | 36.93 (4.20) | ***F* = 5.20**** | Mardi Gras 0 % > All** |
| **Drug Effects Scale** |  |  |  |  |  |  |
| Enjoy | 1.34 (0.17) | 0.41 (0.16) | 1.53 (0.16) | 1.48 (0.16) | *F* = **11.68***** | All > Jamestown*** |
| Crave | 0.91 (0.16) | 0.57 (0.16) | 1.27 (0.16) | 1.33 (0.16) | *F* = 6.05*** | Mardi Gras 0% > Arctic Blast*, Mardi Gras 0% > Jamestown***, Mardi Gras 0% > Jamestown*** |
| Taste | 1.19 (0.17) | 0.37 (0.16) | 1.48 (0.17) | 1.59 (0.16) | *F* = 13.42*** | All > Jamestown*** |
| Like | 1.35 (0.15) | 0.27 (0.15) | 1.26 (0.15) | 1.36 (0.15) | *F* = 13.66*** | All > Jamestown*** |
| Pleasure | 1.26 (0.15) | 0.44 (0.15) | 1.23 (0.15) | 1.02 (0.15) | *F* = 6.55*** | All > Jamestown** |
| **MNWS,** Total Score | 2.27 (0.68) | 3.18 (0.67) | 2.80 (0.67) | 1.52 (0.67) | *F* =2.04 |  |
| **QSU-B,** Total Score | 8.36 (1.64) | 8.33 (1.60) | 12.03 (1.61) | 3.88 (1.60) | ***F* = 6.67***** | All > Mardi Gras 0%*, Mardi Gras 1.2% >Arctic Blast* & Jamestown* |

QSU-B = Questionnaire of Smoking Urges. MNWS = Minnesota Nicotine Withdrawal Scale. All values are mean (standard error). * *p*<.05, ** *p*<.01, *** *p*<.001. “All” refers to all other conditions.
